# Supplementary figures and images for: A genomic-clinical nomogram predicting recurrence-free survival for patients diagnosed with hepatocellular carcinoma
Source: PeerJ. 2019 Oct 31;7:e7942. doi: 10.7717/peerj.7942 (PMC6825747; doi:10.7717/peerj.7942)

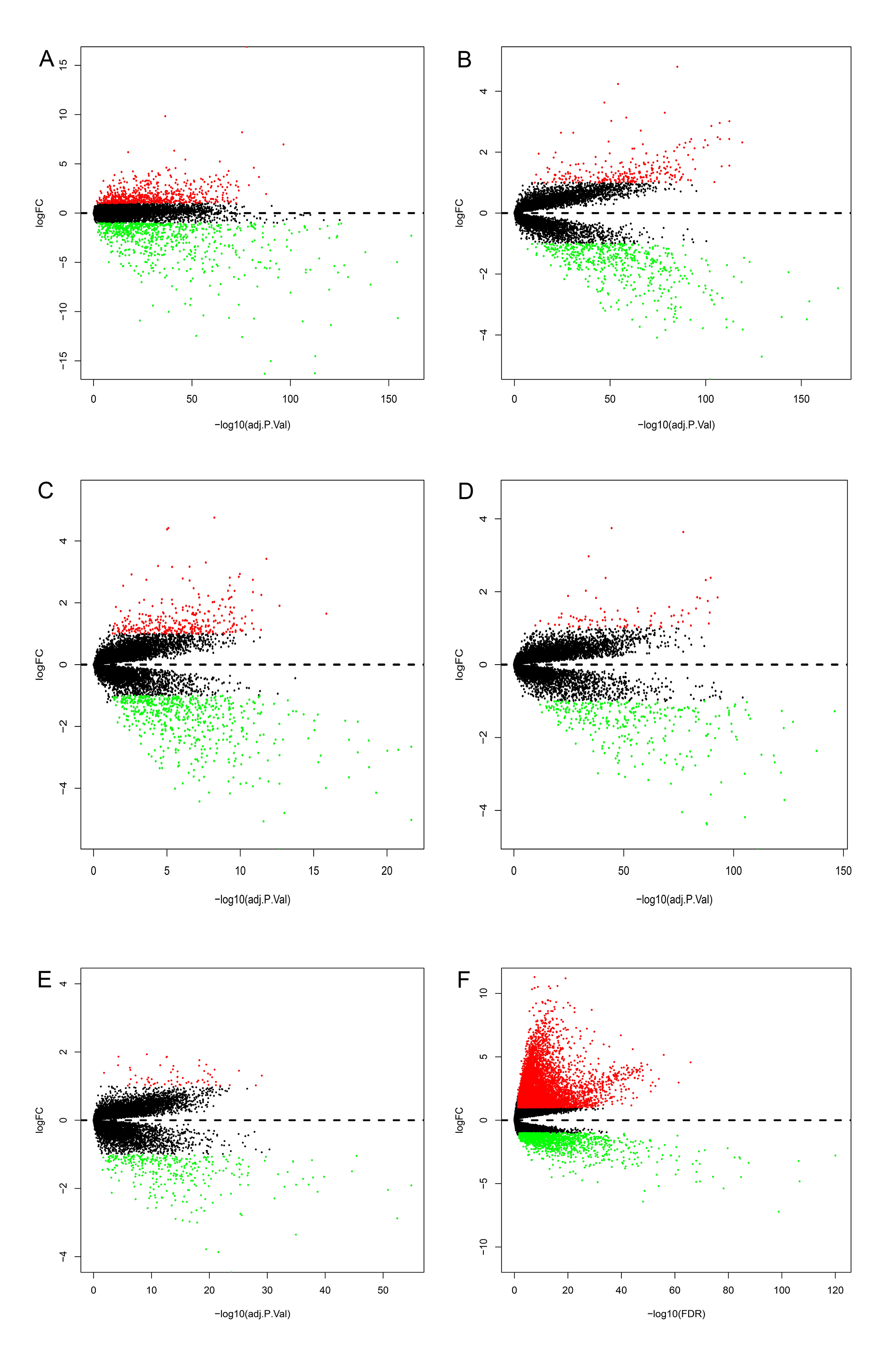

Supplement: Supplemental Information 1 — (A) GSE25097; (B) GSE14520 (platform GPL3921); (C) GSE14520 (platform GPL571); (D) GSE36376; (E) GSE76427; (F) TCGA. Differently expressed genes, DEGs; the Gene Expression Omnibus, GEO; The Cancer Genome Atlas, TCGA. [file peerj-07-7942-s001.png]

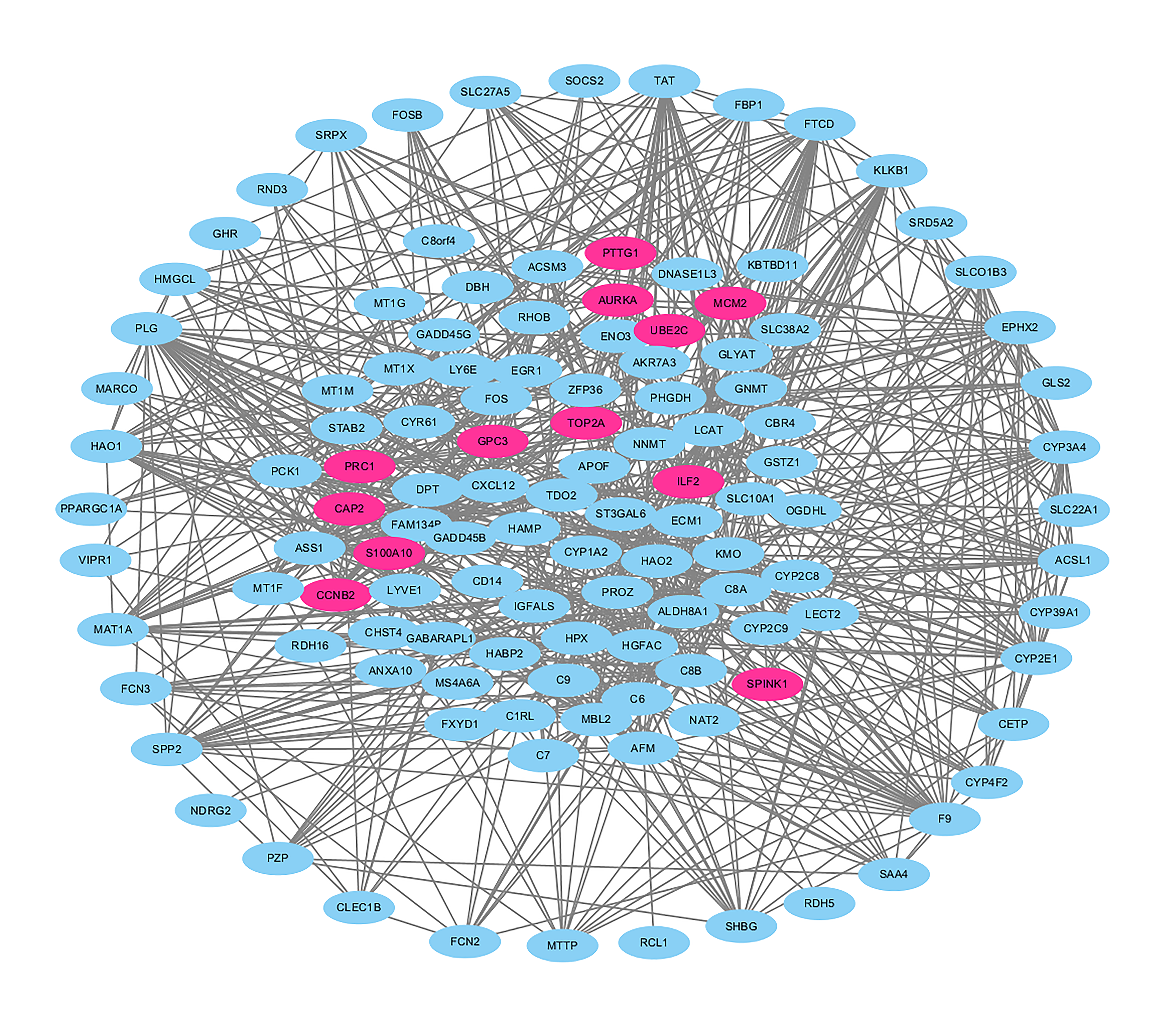

Supplement: Supplemental Information 2 — A PPI network complex containing 121 nodes and 773 edges was constructed to visual the correlations among DEGs. The red nodes represented the proteins of upregulated genes and the blue nodes represented the proteins of downregulated genes. Protein–protein interaction, PPI; Differently expressed genes, DEGs. [file peerj-07-7942-s002.png]

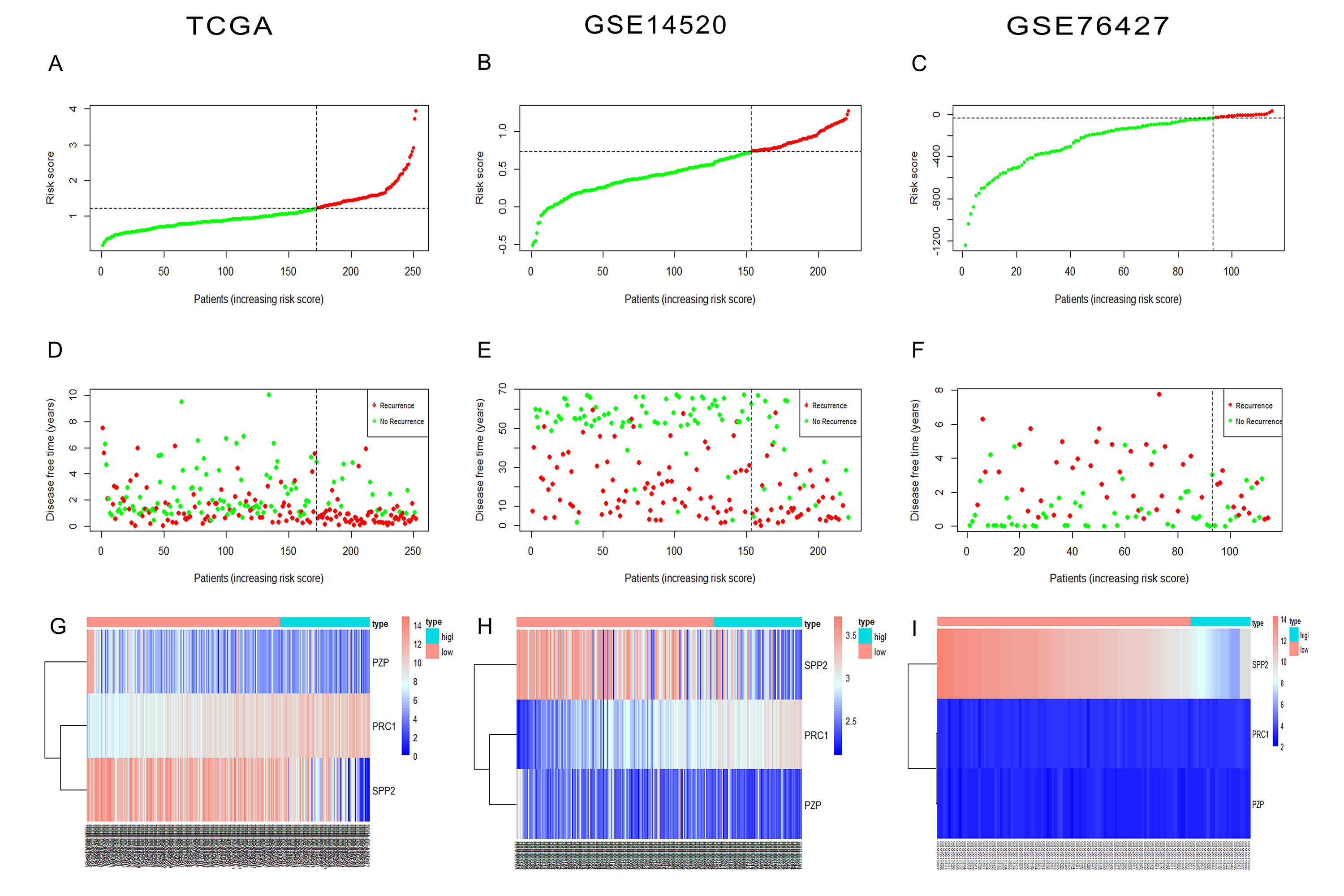

Supplement: Supplemental Information 3 — (A–C) Risk score distribution of HCC patients, (D–F) status of every patient and (G–I) expression heatmap of the three RRGs corresponding to each patient above in TCGA, GSE14520 and GSE76427 datasets. (D–F) Red points represented recurrence and green points represented disease free. Hepatocellular carcinoma, HCC; recurrence-related genes, RRGs; The Cancer Genome Atlas, TCGA. [file peerj-07-7942-s003.png]
