# Supplementary material for: A genomic-clinical nomogram predicting recurrence-free survival for patients diagnosed with hepatocellular carcinoma
Source: PeerJ. 2019 Oct 31;7:e7942. doi: 10.7717/peerj.7942 (PMC6825747; doi:10.7717/peerj.7942)
Supplement: Supplemental Information 4 — NOTE: hepatocellular carcinoma, HCC; the Gene Expression Omnibus, GEO. [file peerj-07-7942-s004.docx]

**Table S1. Details of selected gene expression profiles of HCC from the GEO database**

| Dataset | Year | Country | Author | Platform | Sample number | |
| --- | --- | --- | --- | --- | --- | --- |
|  |  |  |  |  | Tumor | Non-tumor |
| GSE25097 | 2010 | The United States | Zhang et al. | GPL10687 | 268 | 289 |
| GSE14520 | 2009 | The United States | Wang et al. | GPL571 | 22 | 21 |
|  |  |  |  | GPL3921 | 225 | 220 |
| GSE36376 | 2012 | South Korea | Park et al. | GPL10558 | 240 | 193 |
| GSE76427 | 2015 | Singapore | Yenamandra et al. | GPL10558 | 115 | 52 |

NOTE: hepatocellular carcinoma, HCC; the Gene Expression Omnibus, GEO
